# *Strongyloides stercoralis* *age-1*: A Potential Regulator of Infective Larval Development in a Parasitic Nematode

**DOI:** 10.1371/journal.pone.0038587

**Published:** 2012-06-06

**Authors:** Jonathan D. Stoltzfus, Holman C. Massey, Thomas J. Nolan, Sandra D. Griffith, James B. Lok

**Affiliations:** 1 Department of Pathobiology, University of Pennsylvania School of Veterinary Medicine, Philadelphia, Pennsylvania, United States of America; 2 Department of Biostatistics and Epidemiology, University of Pennsylvania Perelman School of Medicine, Philadelphia, Pennsylvania, United States of America; New England Biolabs, United States of America

## Abstract

Infective third-stage larvae (L3i) of the human parasite *Strongyloides stercoralis* share many morphological, developmental, and behavioral attributes with *Caenorhabditis elegans* dauer larvae. The ‘dauer hypothesis’ predicts that the same molecular genetic mechanisms control both dauer larval development in *C. elegans* and L3i morphogenesis in *S. stercoralis*. In *C. elegans*, the phosphatidylinositol-3 (PI3) kinase catalytic subunit AGE-1 functions in the insulin/IGF-1 signaling (IIS) pathway to regulate formation of dauer larvae. Here we identify and characterize *Ss-age-1*, the *S. stercoralis* homolog of the gene encoding *C. elegans* AGE-1. Our analysis of the *Ss-age-1* genomic region revealed three exons encoding a predicted protein of 1,209 amino acids, which clustered with *C. elegans* AGE-1 in phylogenetic analysis. We examined temporal patterns of expression in the *S. stercoralis* life cycle by reverse transcription quantitative PCR and observed low levels of *Ss-age-1* transcripts in all stages. To compare anatomical patterns of expression between the two species, we used *Ss-age-1* or *Ce-age-1* promoter*::enhanced green fluorescent protein* reporter constructs expressed in transgenic animals for each species. We observed conservation of expression in amphidial neurons, which play a critical role in developmental regulation of both dauer larvae and L3i. Application of the PI3 kinase inhibitor LY294002 suppressed L3i *in vitro* activation in a dose-dependent fashion, with 100 µM resulting in a 90% decrease (odds ratio: 0.10, 95% confidence interval: 0.08–0.13) in the odds of resumption of feeding for treated L3i in comparison to the control. Together, these data support the hypothesis that *Ss-age-1* regulates the development of *S. stercoralis* L3i via an IIS pathway in a manner similar to that observed in *C. elegans* dauer larvae. Understanding the mechanisms by which infective larvae are formed and activated may lead to novel control measures and treatments for strongyloidiasis and other soil-transmitted helminthiases.

## Introduction

Helminth infections represent a vast global burden of disease, with parasitic nematodes infecting more than one billion people [1], [2]. The infectious form of many parasitic nematodes, including the medically relevant species which cause strongyloidiasis, filariasis, and hookworm disease [3], is the third-stage larva (L3i). L3i developmentally arrest, sometimes for months, before resuming development upon encountering a host [3], [4]. Despite their potential as new therapeutic targets, the signaling proteins and pathways controlling developmental arrest and activation of L3i are unknown.

Our lab has employed *Strongyloides stercoralis*, a parasitic nematode infecting nearly 100 million people [2], [5], as a model to study molecular mechanisms in parasitic nematodes, and we have developed methods for transgenesis that are unavailable in other species [6]–[8]. Study of *S. stercoralis* is facilitated by a life cycle that includes both parasitic and free-living generations, allowing us to probe factors driving L3i development in an otherwise “obligately parasitic" group of organisms [8], [9]. In *S. stercoralis*, L3i form conditionally in the post-parasitic generation (homogonic development), along with free-living males and females (heterogonic development), and constitutively in the post-free-living generation [10]. This life cycle provides a unique opportunity to interrogate the mechanisms governing L3i formation and activation.

A similar developmentally arrested stage, the dauer larva, is formed by the free-living nematode *Caenorhabditis elegans* in response to unfavorable conditions [11], [12]. Interestingly, *C. elegans* dauer larvae and *S. stercoralis* L3i arrest at the same third larval stage and share similar characteristics of morphology, extended lifespan, stress-resistance, and cessation of feeding [13], [14]. Dauer larvae and L3i resume development soon after encountering favorable environmental conditions or the definitive host, respectively [10], [15]. The “dauer hypothesis" proposes that the molecular mechanisms governing L3i developmental arrest and recovery in *S. stercoralis* and other parasitic nematodes are similar to those regulating dauer formation and recovery in *C. elegans* [12], [14], [16], [17].

In *C. elegans*, the insulin/IGF-1 signaling (IIS) pathway plays a critical role in dauer formation and recovery. When environmental conditions are limiting, IIS signaling is suppressed, resulting in dauer arrest [18]. In response to favorable environmental cues [19], [20], the IIS pathway is activated and promotes continuous growth and development. When IIS is activated via a muscarinic agonist [21], insulin-like peptides are secreted [22], [23] and bind the insulin-like receptor DAF-2 [18]. DAF-2 agonists [22]–[25] activate the phosphatidylinositol-3 (PI3) kinase [26], a heterodimer composed of an AGE-1 catalytic subunit [27] and an AAP-1 accessory/regulatory subunit [28]. PI3 kinase activation triggers a signaling cascade resulting in phosphorylation of the forkhead transcription factor DAF-16, which ultimately causes its removal from the nucleus [29], [30]. Loss-of-function alleles for both *daf-2* and *age-1* result in a constitutive dauer arrest phenotype, while *daf-16* mutations result in a dauer defective phenotype, demonstrating the importance of IIS for regulating dauer development [31].

Members of the IIS pathway have been cloned from several parasitic nematodes [32]–[34], including *S. stercoralis*, but it remains unclear whether they comprise a pathway controlling the developmental arrest and activation of L3i. Recent studies in our lab have demonstrated that the *S. stercoralis* DAF-16 homolog is required for normal arrest of L3i under conditions of decreased IIS [6]; however, it is unknown whether the IIS pathway also regulates developmental activation of L3i in the host through increased IIS. Since *Ce-age-1* is the main mediator of increased IIS signaling from the *Ce-daf-2* insulin-like receptor to the downstream *Ce-daf-16* forkhead transcription factor [35], we endeavored to clone and characterize the *S. stercoralis* homolog of *Ce-age-1*. Here we present striking similarities between *Ce-age-1* and *Ss-age-1* as well as the first evidence of IIS regulating L3i activation in *S. stercoralis*.

## Materials and Methods

### Ethics Statement

No human subjects were used in these studies. The *S. stercoralis* UPD strain and PV001 line were maintained in prednisolone-treated beagles in accordance with protocols 702342, 801905, and 802593 approved by the University of Pennsylvania Institutional Animal Care and Use Committee (IACUC). Experimental infections of *S. stercoralis* were conducted in Mongolian gerbils under the same IACUC-approved protocols, and animals were sacrificed by CO_2_ asphyxia in accordance with standards established by the American Veterinary Medical Association. All IACUC protocols, as well as routine husbandry care of the animals, were carried out in strict accordance with the *Guide for the Care and Use of Laboratory Animals of the National Institutes of Health*.

### *S. stercoralis* and *C. elegans* Strains and Maintenance

The *S. stercoralis* UPD strain was maintained and cultured as previously described [8], [36], [37]. UPD strain free-living adults for DNA transformation and RNA extraction were isolated via the Baermann technique from two-day-old charcoal coprocultures incubated at 22°C. UPD strain L3i for genomic DNA and *in vitro* activation were isolated via the Baermann technique from seven-day-old charcoal coprocultures incubated at 25°C.

The *Strongyloides stercoralis* isofemale line PV001 was derived from the UPD strain as a line with limited genetic variability. To derive this line, virgin free-living females were prepared by placing one first-stage larva, freshly isolated from the feces of a dog infected with the UPD strain, into each well of a 96 well tissue culture plate. The plate had been prepared by placing 50 µl of 1% agar (Lonza, Basel, Switzerland) into each well and then adding a small (several mg) piece of normal dog feces to the surface. After three days, two of the 96 wells contained an adult female and larval progeny. The mechanism by which progeny arise in the absence of mating in the free living generation is unknown. However, *S. stercoralis* parasitic females are presumed to reproduce by mitotic parthenogenesis [38], [39], and it is possible that this phenomenon occurs at a low frequency among free-living females as well. At six days post plating, 34 L3i were removed from one of the positive wells and used to infect a gerbil. The gerbil was given 2 mg methylprednisolone acetate (SQ) at the time of infection and weekly thereafter [37]. L3i recovered from coprocultures made from this gerbil’s feces were used to infect another gerbil and the L3i recovered from coprocultures of this second gerbil’s feces were used to infect the dog from which feces were isolated for use in the RT-qPCR study.

*C. elegans* N2 worms were maintained by standard methods [40]. The CY246 strain, *age-1(mg44); sqt-1(sc13)/mnC1 dpy-10(e128); unc-52(e444)* II, a gift from Catherine Wolkow, was maintained by picking phenotypically wild-type (WT) worms. The *age-1^−/−^* F1 progeny in this strain, which are marked in *cis* with the *sqt-1* roller phenotype and exhibit maternal rescue, produce F2 progeny that constitutively form roller dauers. The BC10837 strain [41], *dpy-5(e907)* I, sEx10837 [rCesB0334.8::GFP + pCeh361], was obtained from the *Caenorhabditis* Genetics Center (University of Minnesota, Minneapolis, Minnesota, USA) and maintained by picking GFP-positive phenotypically WT worms.

### DNA and cDNA Preparation

Genomic DNA from the *S. stercoralis* UPD strain was isolated from L3i by phenol/chloroform extraction [7]. UPD strain RNA for cDNA synthesis was isolated from free-living adults by TRIzol reagent extraction (Life Technologies, Grand Island, New York, USA); total cDNA was synthesized using SuperScript III reverse transcriptase and an oligo dT primer (Life Technologies) following the manufacturer’s protocol. cDNA with ligated adapters for 5′ and 3′ RACE was prepared using the GeneRacer kit (Life Technologies).

For *C. elegans* genomic DNA preparations, starved plates of N2 worms were washed and DNA extracted using the DNeasy Blood and Tissue kit (Qiagen, Valencia, California, USA). RNA for cDNA synthesis was isolated from mixed-stage plates of N2 worms and cDNA synthesized by the same methods as for *S. stercoralis*.

### Cloning of *S. stercoralis* Genes

The genomic sequence of *Ss-age-1* was determined using degenerate primers to amplify a coding sequence homologous to a *Parastrongyloides trichosuri* EST (GenBank: BI742945), which was identified by BLAST searches of the nematode EST database (http://nematode.net/NN3_frontpage.cgi?navbar_selection=nemagene&subnav_selection=nemablast) with the *Ce*-AGE-1 sequence (GenBank: AAC47459). Repeated cycles of inverse PCR and established methods were used to determine the complete coding sequence as well as sequences of the 5′ and 3′ regions of *Ss-age-1* (GenBank: JQ772018) [32], [42], [43]. Both 5′ and 3′ RACE were performed using the GeneRacer protocol (Life Technologies) and Phusion polymerase (New England BioLabs, Ipswich, Massachusetts, USA). 5′ RACE used the Ss-age-1-17R outer primer and Ss-age-1-20R nested primer; 3′ RACE used the Ss-age-1-20F outer primer and Ss-age-1-11F nested primer (Table S1). Genomic DNA and RACE PCR products were cloned using the Zero Blunt TOPO PCR cloning kit (Life Technologies) and sequenced in triplicate. The full-length *Ss-age-1* cDNA was amplified from total cDNA with Phusion polymerase (New England BioLabs) using the Ss-age-1-24FattB1 and Ss-age-1-24RstopattB2 primers, while 1.3 kb of 5′ region was amplified using the Ss-age-1-23FattB4 and Ss-age-1-23RattB1r primers (Table S1). The 1,327 bp 5′ region (pPV450) and 3,630 bp *Ss-age-1* cDNA (pPV451) were cloned into pDONR P4-P1R and pDONR221, respectively, using Gataway recombination (Life Technologies).

The genomic sequence of *Ss-aap-1* (GenBank: JQ781500) was PCR amplified using degenerate primers for the *Strongyloides ratti* sequence, identified by BLAST searches of the *S. ratti* genome database (http://www.sanger.ac.uk/resources/downloads/helminths/strongyloides-ratti.html) with the *Ce*-AAP-1 (NCBI: NM_059121) sequence. This PCR product was cloned into pCR-Blunt II-TOPO and sequenced. Similar to *Ss-age-1*, 5′ RACE of *Ss-aap-1* was performed using the Ssaap1-2R outer primer and Ssaap1-3R nested primer (Table S1), with the product cloned into pCR-Blunt II-TOPO and sequenced in triplicate.

For both *Ss*-AGE-1 and *Ss*-AAP-1, protein domains were determined using BLASTp searches through NCBI’s Conserved Domain Database service (http://blast.ncbi.nlm.nih.gov/Blast.cgi) [44]. Protein coding sequences for *S. ratti age-1* and *aap-1* homologs were identified by manual annotation of the respective *S. ratti* contigs. The close homology of the predicted peptides (Dataset S1) and conservation of introns between *S. ratti* and *S. stercoralis* aided this process.

### Phylogenetic Analysis

A protein alignment of PI3 kinase catalytic subunits was generated using Clustal X2 [45] and a BLOSUM62 matrix. A neighbor-joining tree was constructed using MEGA version 4.0 [46] with bootstrapping scores (1000 iterations) shown for robust clades (>50% cutoff). A global protein alignment between *Ce*-AGE-1 and *Ss*-AGE-1 was performed using EMBOSS Needle and a BLOSUM62 matrix [47] and was annotated for coding exons to determine conservation of introns between *Ss-age-1* and *Ce-age-1*. Accession numbers were: *Strongyloides stercoralis* (GenBank: JQ772018), *Strongyloides ratti* (Dataset S1), *Parastrongyloides trichosuri* AGE-1 (GenBank: ADN44511), *Brugia malayi* PI3K (NCBI: XP_001902593), *Caenorhabditis briggsae* AGE-1 (NCBI: XP_002631094), *Caenorhabditis elegans* AGE-1 (GenBank: AAC47459), *Homo sapiens* PI3KCA (GenBank: AAI13604), *Drosophila melanogaster* Pi3K92E (NCBI: NP_650902), *Drosophila melanogaster* Pi3K59F (NCBI: NP_477133), *Homo sapiens* PI3KC3 (NCBI: NP_002638), *Caenorhabditis elegans* VPS-34 (NCBI: NP_491741), *Saccharomyces cerevisiae* VPS34 (NCBI: NP_013341), *Drosophila melanogaster* Pi3K68D (NCBI: NP_524028), *Caenorhabditis elegans* F39B1.1 (NCBI: NP_510529), and *Homo sapiens* PI3KC2A (NCBI: NP_002636).

A protein alignment of PI3 kinase accessory/regulatory subunits using Clustal W and a BLOSUM matrix was generated using Geneious version 5.5.6 [48] and was annotated for coding exons to determine conservation of introns between *Ss-aap-1* and *Ce-aap-1*. A global protein alignment between *Ce*-AAP-1 and *Ss*-AAP-1 was performed using EMBOSS Needle and a BLOSUM62 matrix [47]. Accession numbers were: *Strongyloides stercoralis* AAP-1 (GenBank: JQ781500), *Strongyloides ratti* AAP-1 (Dataset S1), *Caenorhabditis elegans* AAP-1 (NCBI: NM_059121), *Trichinella spiralis* AAP-1 (GenBank: EFV56516), *Brugia malayi* AAP-1 (GenBank: EDP31759), *Ascaris suum* AAP-1 (GenBank: ADY45992), *Drosophila melanogaster* PI3K-21b (GenBank: CAA73100), and *Homo sapiens* PI3K-p85a (UniProtKB: P27986).

### RT-qPCR

Developmental stages of *S. stercoralis* line PV001 were isolated for RT-qPCR as previously described [32], washed, and rendered free of fine particle debris by migration through agarose [49] into BU buffer [40]. See Supplemental Methods for detailed protocol (Text S1). Worms were snap-frozen in TRIzol reagent (Life Technologies) in liquid nitrogen; total RNA was extracted using the manufacturer’s protocol. Purified RNA was quantified using the Bioanalyzer 2100 (Agilent Technologies, Inc., Santa Clara, California, USA) and only samples with an RNA integrity number greater than 8.0 were used.

Gene specific primer sets, which only amplified spliced cDNA, included Ss-age-1RT-3F and -R, Ss-act2RT-2F and -R, and Ss-gapdhRT-2F and -R (Table S1) [7], [50], [51]. Primer sets were calibrated using a five dilution series of total RNA and efficiencies calculated using standard methods [52]. Gene specific RT-qPCR was performed on three biological replicates, each in duplicate, using the Brilliant II SYBR Green QRT-PCR kit (Agilent Technologies) with 50 ng of total RNA on an Applied Biosystems 7500 (Life Technologies) instrument with the following parameters: RT step of 30 min at 50°C; 10 min at 95°C; and 40 cycles of 30 sec at 95°C and 60 sec at 60°C (detection step); followed by a melting temperature curve. Controls omitting template or reverse transcriptase were also included.

Results were analyzed by computing the mean Ct value for each replicate. Abundances were calculated using the slope of the calibration curve and normalizing to an arbitrarily determined mean of 10 copies of *Ss-age-1* in free-living females [51], [52]. Log transformed values, +1 SEM, were plotted in Prism version 5.03 (GraphPad Software, Inc., La Jolla, California, USA).

### Plasmid Construction

Plasmids containing *Ce-age-1* promoter and *egfp* reporter constructs were assembled by overlap extension PCR. The construct *Ce-age-1p::egfp::Ce-tbb-2t*, contained in plasmid pPV452, was assembled in two steps. The 866 bp of *Ce-age-1* 5′ region was PCR amplified using *C. elegans* genomic DNA as a template. The 870 bp *egpf* coding region, including introns, and 333 bp of the *Ce-tbb-2* 3′ region (terminator) were amplified using pJA257 as template (Addgene, Cambridge, Massachusetts, USA). These PCR products were fused by overlap extension PCR using the overlapping primers Ce-age-1-EGFP-F and -R (Table S1). The product was cloned into pUC19 (New England Biolabs) and the insert fully sequenced.

The construct *Ce-age-1p::Ce-age-1(102bp)::egfp::Ce-age-1t*, contained in plasmid pPV455, was assembled by a similar scheme. The 919 bp segment of *Ce-age-1* 5′ region, including the first 102 bp of the *Ce-age-1* coding sequence, was PCR amplified using *C. elegans* genomic DNA as a template. The 870 bp *egfp* coding sequence, with introns, was PCR amplified using pJA257 as a template. A 1,071 bp segment of *Ce-age-1* 3′ region (terminator) was PCR amplified using genomic DNA as a template. These three segments were joined using the overlapping primers Ex10837-EGFP-F and -R and EGFP-Ceage1t-F and -R (Table S1), the product T/A cloned into the pCR-TOPO-XL vector (Life Technologies), and the insert fully sequenced.

The construct *Ce-age-1p::Ce-age-1(3,549bp)::Ce-unc-54t*, contained in plasmid pPV454, was assembled by a similar process. An 873 bp segment of *Ce-age-1* 5′ region was amplified from genomic DNA and fused to 2,376 bp of the 5′ region of *Ce-age-1*, amplified from cDNA, using the overlapping primers Ceage1p-cDNA-F and -R (Table S1). This was subsequently fused to the remainder of the *Ce-age-1* cDNA with the plasmid Pdpy-30-age-1, a gift from Yuichi Iino [53], as template and using the overlapping primers Ceage1-Cat-F and -R (Table S1). Finally, the promoter and full-length 3,549 bp *Ce-age-1* cDNA were fused to 765 bp of the *Ce-unc-54* 3′ region (terminator), amplified from Pdpy-30-age-1 template, using the overlapping primers Ceage1-unc54-F and -R (Table S1). The PCR product was T/A cloned into the pCR4-TOPO vector (Life Technologies) and the insert fully sequenced.

The construct *Ss-age-1p::egfp::Ss-era-1t*, contained in plasmid pPV453, was assembled by a similar process. A 1,326 bp segment of *Ss-age-1* 5′ region was amplified from genomic DNA. The 870 bp *egfp* coding sequence, including introns, was amplified from pJA257. A 603 bp segment of *Ss-era-1* 3′ region (terminator) was amplified from pAJ04 (Addgene). These three segments were fused using the overlapping primers Ss-age-1-EGFP-F and -R and EGFP-Ss-era-1-F and -R (Table S1) and cloned into pCR-Blunt II-TOPO with the insert fully sequenced.

### Transformation of *C. elegans* and *S. stercoralis*

*C. elegans* N2 (WT) or CY246 (phenotypically WT) adult hermaphrodites were transformed by gonadal microinjection using standard techniques [54]. Plasmids were injected at a concentration of 20 ng/µl for *Ce-age-1p::egfp::Ce-tbb-2t* (pPV452) and *Ce-age-1p::Ce-age-1(102bp)::egfp::Ce-age-1t* (pPV455) along with 80 ng/µl non-coding pUC19 DNA. Transformations with *Ce-age-1p::Ce-age-1(3,549bp) ::Ce-unc-54t* (pPV454) at 20 ng/µl included the *Ce-myo-2p::mCherry::Ce-unc-54t* (pCFJ90) (Addgene) co-injection marker at 2 ng/µl and 78 ng/µl pUC19. Injected hermaphrodites were transferred to NGM+OP50 plates and progeny screened for fluorescence. Lines were established from single transformed F2 worms. The CY246+pPV454+pCFJ90 lines were assessed for dauer rescue by presence/absence of progeny from F1 rollers. The F2 roller progeny were subsequently passaged for more than 10 generations. Two lines of CY246 transformed with the co-injection marker pCFJ90, injected at 2 ng/µl and 98 ng/µl of pUC19, were used as a control.

*S. stercoralis* was transformed by gonadal micro-injection of adult free-living females as previously described [8]. A mix of 40 ng/µl *Ss-age-1p::egfp::Ss-era-1t* (pPV453) and 20 ng/µl *Ss-act-2::mRFPmars::Ss-era-1t* (pAJ50; Addgene) [7] was injected in females, which were then paired with an excess of adult males on an NGM+OP50 plate and incubated at 22°C. The F1 progeny were screened for fluorescence 48 and 72 hours after micro-injection.

*C. elegans* and *S. stercoralis* larvae were screened for expression of fluorescent reporter transgenes using an Olympus SZX12 stereomicroscope with coaxial epifluorescence. Transgenic *C. elegans* and *S. stercoralis* worms were examined in detail using an Olympus BX60 compound microscope equipped with Nomarski Differential Interference Contrast (DIC) optics and epifluorescence (Olympus America Inc., Center Valley, Pennsylvania, USA). Specimens were immobilized on a 2% agarose pad (Lonza), anesthetized using 10 mM levamisole (Sigma-Aldrich), and imaged with a Spot RT Color digital camera and Spot Advanced image analysis software (Diagnostic Instruments, Inc., Sterling Heights, Michigan, USA). Captured images were processed using GIMP version 2.6 (www.gimp.org).

### *S. stercoralis* L3i in vitro Activation

*In vitro* activation of *S. stercoralis* L3i was performed as previously described [55], [56] with the following adaptations. All conditions were supplemented with antibiotics (final concentration: 100 U/ml penicillin, 10 µg/ml streptomycin, and 12.5 µg/ml tetracycline). M9 buffer was used as the medium for the negative control [40].

DMEM (supplemented with L-glutamine, 4.5 g/L glucose, and sodium pyruvate) was used as the basal medium for the positive control and experimental conditions. The dimethyl sulfoxide (DMSO) (carrier) positive control and all experimental conditions had a final concentration of 10% naïve canine serum, 12.5 mM reduced glutathione (Sigma-Aldrich, St. Louis, Missouri, USA), and 1.3% DMSO (Sigma-Aldrich). Four concentrations of LY294002 (CAS registry number: 154447-36-6) (LC Laboratories, Woburn, Massachusetts, USA) were assessed: 500 µM, 100 µM, 50 µM, and 10 µM.

For the *in vitro* activation assay, L3i were isolated from seven-day-old charcoal coprocultures by the Baermann technique at 26°C. L3i were incubated in 100 µl aliquots of culture media at 37°C and 5% CO_2_ for 21 hours, with three replicates for each condition. Cultures were then spiked with 2.5 µl fluorescein isothiocyanate (FITC) (20 mg/ml in dimethylformamide) and incubated an additional three hours for a total of 24 hours. L3i for each condition were pooled and washed five times in M9 buffer with low speed centrifugation. L3i were then mounted on glass slides with grease edged cover-slips, immobilized by a 15 second heat-shock at 60°C, and viewed by fluorescence microscopy. Only L3i with FITC in the pharynx were scored as “positive" for feeding.

Due to toxicity at 500 µM LY294002, resulting in greater than 50% death of L3i, this condition was discarded from the analysis. For all other conditions, greater than 90% of L3i were active at 24 hours. Data for five experiments were plotted, +1 SEM, in Prism version 5.03 (GraphPad Software, Inc.). The relationship between condition and resumption of feeding was modeled using a generalized linear mixed-effects model and a logistic regression model, with condition as either a categorical predictor or a linear predictor. A logistic regression model with condition as a categorical predictor best described the data and was used to calculate the odds ratios, 95% confidence intervals, and p-values for each condition with respect to the DMSO control. All statistical models were fit using R version 2.14.1 [57].

## Results and Discussion

### Identification of *S. stercoralis* Age-1

**Figure 1:**
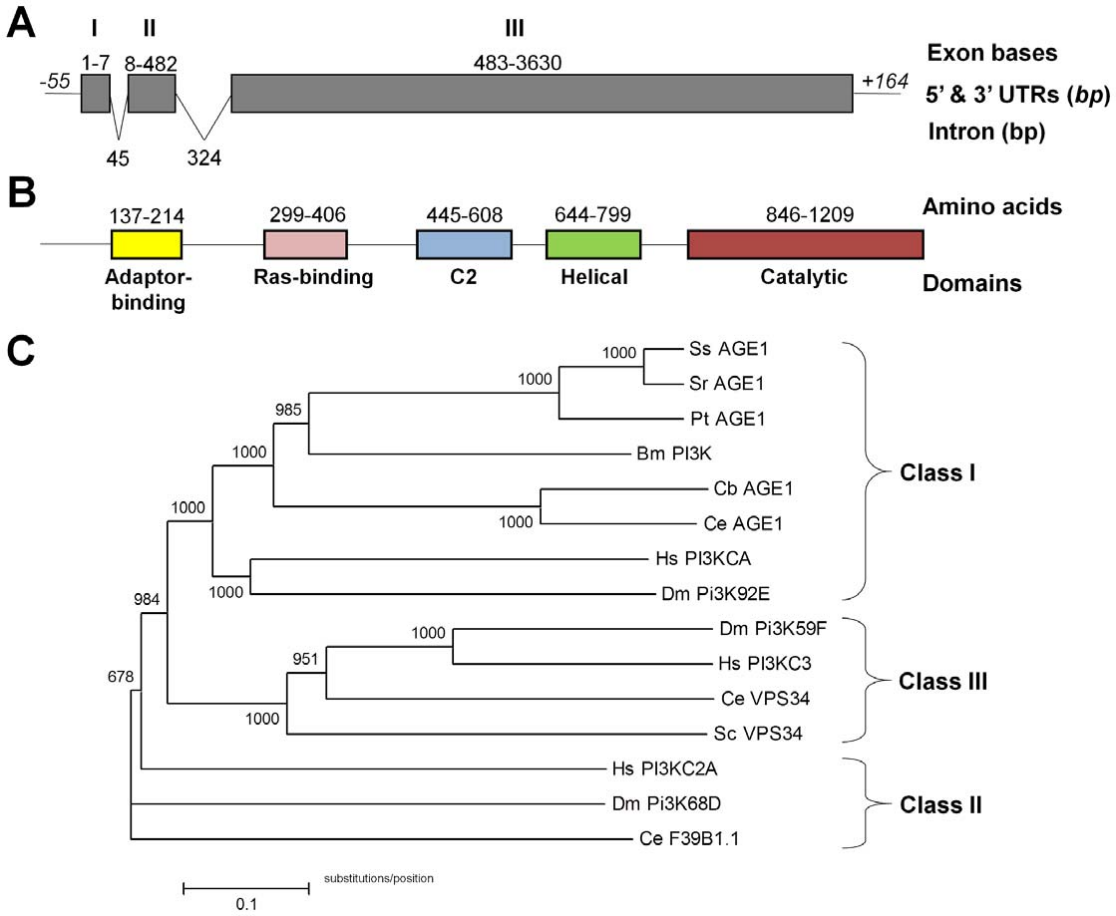
*Ss*-AGE-1 is a homolog of the *Ce*-AGE-1 PI3 kinase catalytic subunit. (A) Intron-exon structure of the *Ss-age-1* unspliced mRNA sequence. Grey boxes indicate the three exons, with the numbers above indicating the first and last base pairs of the exon. Introns are indicated by slanted lines between the exons, with the numbers indicating the total intron length. The 5′ and 3′ untranslated regions (UTR) are indicated with horizontal lines, with the italicized numbers indicating the total length of the UTR in base-pairs (bp). (B) Domain structure of the *Ss*-AGE-1 predicted protein. Shaded boxes represent the five protein family domains, with the numbers indicating the first and last amino acids of the domain. (C) Phylogenetic analysis of *Ss*-AGE-1. The predicted *Ss*-AGE-1 protein groups with other class I PI3 kinase catalytic subunits, including *Ce*-AGE-1. Abbreviations: *Strongyloides stercoralis* (Ss), *Strongyloides ratti* (Sr), *Parastrongyloides trichosuri* (Pt), *Brugia malayi* (Bm), *Caenorhabditis briggsae* (Cb), *Caenorhabditis elegans* (Ce), *Homo sapiens* (Hs), *Drosophila melanogaster* (Dm), and *Saccharomyces cerevisiae* (Sc). Accession numbers listed in Methods.

To identify the *S. stercoralis* homolog of *Ce-age-1*, we performed BLAST searches of the nematode expressed sequence tag (EST) database and found an EST with homology to *Ce-age-1* in *Parastrongyloides trichosuri*, a closely related parasitic nematode. We designed degenerate primers and amplified an *S. stercoralis* sequence from genomic DNA. Using successive rounds of inverse PCR, we elucidated the 3,999 base-pair (bp) genomic coding region for *Ss-age-1* along with 1.3 kilobase (kb) of the upstream sequence and 1.8 kb of the downstream sequence (GenBank: JQ772018). To determine the 5′ and 3′ ends of the *Ss-age-1* coding sequence, we performed rapid amplification of cDNA ends (RACE) using adapter-ligated cDNA. Subsequently, we cloned the 3,630 bp *Ss-age-1* coding sequence from cDNA derived from free-living adults (Figure 1A) and inferred a predicted peptide of 1,209 amino acids (Figure 1B). The locations of the three introns in *Ss-age-1* were not conserved in *Ce-age-1*.

Since *S. stercoralis* and *C. elegans* are members of nematode clades IV and V, respectively, their common ancestry is much more distant than their morphologic similarity suggests [58]. Therefore, we performed phylogenetic analysis to determine whether the predicted *Ss*-AGE-1 protein grouped with *Ce*-AGE-1. We obtained catalytic subunit sequences from public databases for class I, II, and III PI3 kinases found in a variety of metazoan organisms [59]. The predicted proteins were aligned, and a neighbor-joining phylogenetic tree was constructed (Figure 1C). In this analysis, *Ss*-AGE-1 grouped with other class I PI3 kinase catalytic subunits, including *Ce*-AGE-1. A global protein alignment of *Ss*-AGE-1 and *Ce*-AGE-1 demonstrated an overall 30.6% amino acid identity and 49.6% similarity. A search of the conserved domain database also revealed conservation of the five PI3 kinase catalytic subunit domains in *Ss*-AGE-1 [44], [59]. Therefore, we conclude that *Ss-age-1* is indeed the homolog of *Ce-age-1*.

### Identification of *S. stercoralis* aap-1

Functional PI3 kinases include both a catalytic subunit as well as an accessory/regulatory subunit, each encoded by a separate gene [59]. The PI3 kinase accessory/regulatory subunit in *C. elegans* has been identified as *Ce*-AAP-1 [28]. To identify the accessory/regulatory subunit for *Ss*-AGE-1, we BLAST-searched the *Strongyloides ratti* genome database for the homolog of *Ce*-AAP-1. We identified a putative *S. ratti* AAP-1 homolog and used degenerate primers to amplify a genomic region encoding *Ss*-AAP-1 from *S. stercoralis* genomic DNA.

Using the same protocol as for *Ss-age-1*, we performed 5′ RACE and identified the coding sequence of *Ss-aap-1* (GenBank: JQ781500). The location of the single intron in *Ss-aap-1* was not conserved in *Ce-aap-1*. Protein alignment with other PI3 kinase accessory/regulatory subunits revealed close similarity to *Ce*-AAP-1 as well as conservation of both SH2 domains, which mediate binding to phosphorylated tyrosine residues on the activated insulin-like receptor [28], [60] (Figure S1). A global protein alignment of *Ss*-AAP-1 and *Ce*-AAP-1 demonstrated an overall 14.9% amino acid identity and 28.9% similarity. This analysis led us to conclude that the components of a functional PI3 kinase, consisting of an *Ss*-AGE-1 catalytic subunit and an *Ss*-AAP-1 accessory/regulatory subunit, are present in *S. stercoralis*.

### *Ss-Age-1* is Expressed Throughout the *S. stercoralis* Life Cycle

In *C. elegans*, it is hypothesized that regulation of IIS is controlled transcriptionally at the level of the insulin-like peptides, while the cytoplasmic signaling components, including AGE-1, are constitutively expressed. Low, but constitutive, expression of *Ce-age-1* has been noted in microarray analyses in both the various *C. elegans* developmental stages [41] and dauer recovery [61]. A recent study confirmed these findings through a careful quantitative examination of all *C. elegans* insulin-like peptides, *daf-2*, *age-1*, and *daf-16* transcripts in a variety of developmentally arrested and reproductively developing stages [62].

**Figure 2:**
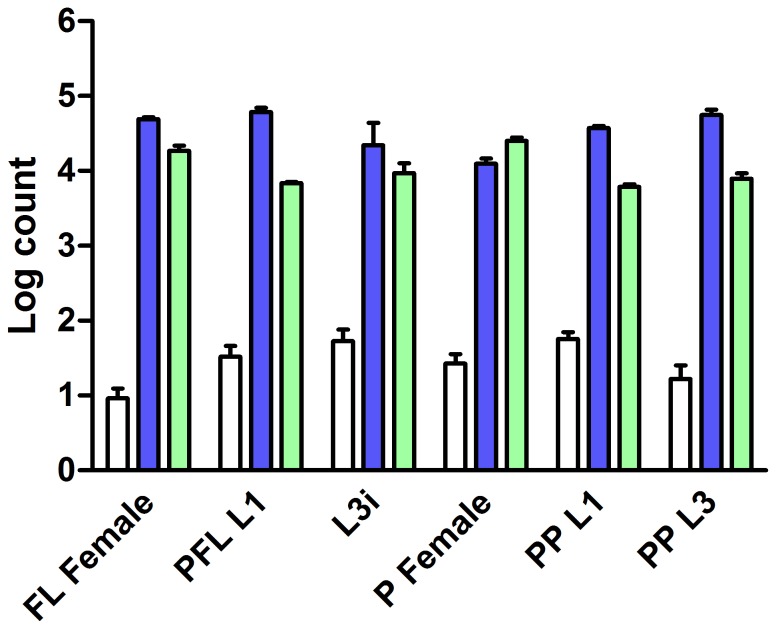
*Ss-age-1* is expressed at a low level in all examined *S. stercoralis* life stages. *Ss-age-1* (white bars) transcript levels in comparison to transcript levels of two reference genes, *Ss-act-2* (dark blue bars) and *Ss-gapdh* (light green bars), in six *S. stercoralis* developmental stages: free-living females (FL Female), post-free-living first-stage larvae (PFL L1), infectious third-stage larvae (L3i), parasitic females (P Female), predominantly (>95%) heterogonically developing post-parasitic first-stage larvae (PP L1), and post-parasitic approximately third-stage larvae heterogonically developing to free-living adults (PP L3). Transcript levels were normalized to *Ss-age-1* in free-living females and log transformed. Error bars represent +1 SEM.

To determine whether *Ss-age-1* is transcriptionally regulated or constitutively expressed over the course of the *S. stercoralis* life cycle, we performed reverse transcription quantitative PCR (RT-qPCR) for six developmental stages. Transcript abundance was calculated for *Ss-age-1*, as well as two reference genes, *actin-2* (*Ss-act-2*) and *glyceraldehyde 3-phosphate dehydrogenase* (*Ss-gapdh*), using 50 ng of total RNA isolated from three biological replicates of each developmental stage. We calculated transcript abundances, which were normalized to an arbitrarily determined mean of 10 copies of *Ss-age-1* in free-living females and log transformed. We observed *Ss-age-1* expression at low, but consistent, levels in comparison to both *Ss-act-2* and *Ss-gapdh* for all life stages examined (Figure 2). Although expression of *Ss-age-1* was at its nadir in free-living females, the biological relevance of this difference is questionable, since similar levels of variability between developmental stages were also observed for *Ss-act-2* and *Ss-gapdh*. Overall, these data suggest that, like *Ce-age-1*, *Ss-age-1* is expressed at a low level throughout the course of the *S. stercoralis* life cycle. This is consistent with the hypothesis that IIS signaling through AGE-1 is regulated post-translationally and not at the transcriptional level.

### *Ce-Age-1* and *Ss-Age-1* have Similar Anatomical Expression Patterns

In *C. elegans*, expression of a *Ce-age-1* cDNA from either a pan-neuronal or intestinal promoter is sufficient to rescue the constitutive dauer arrest phenotype of strong *Ce-age-1* mutant alleles [63]. Thus, it is thought that IIS signaling in amphidial neurons and/or intestinal cells is important for regulating dauer arrest and activation. To determine whether anatomical expression of *Ss-age-1* was similarly regulated, we compared expression of transcriptional reporters under the control of putative promoters for *Ce-age-1* and *Ss-age-1*.

We first sought to determine the tissues in which *Ce-age-1* is expressed to serve as a basis for comparison to *Ss-age-1*. Previous work has reported expression of a *Ce-age-1* transcriptional reporter in amphidial neurons and the intestine, although the sole publicly available image of the BC10837 strain is limited to the head region of the worm [41], [64]. To corroborate and expand these findings, we obtained the BC10837 strain and imaged first-stage larvae (L1) and adult hermaphrodites. In order to replicate the findings from the BC10837 strain, we constructed two transgenic *C. elegans* lines, in the wild-type N2 background, that expressed enhanced green fluorescent protein (EGFP) driven by a *Ce-age-1* promoter with a *Ce-tbb-2* 3′ sequence from extra-chromosomal arrays. To determine whether regulatory sequences for *Ce-age-1* expression were present in the first part of the coding sequence or the 3′ region, three additional lines were transformed with a construct that fused the first 102 bp of *Ce-age-1* coding sequence to *egfp*, driven by the *Ce-age-1* promoter and using the native *Ce-age-1* 3′ sequence.

**Figure 3:**
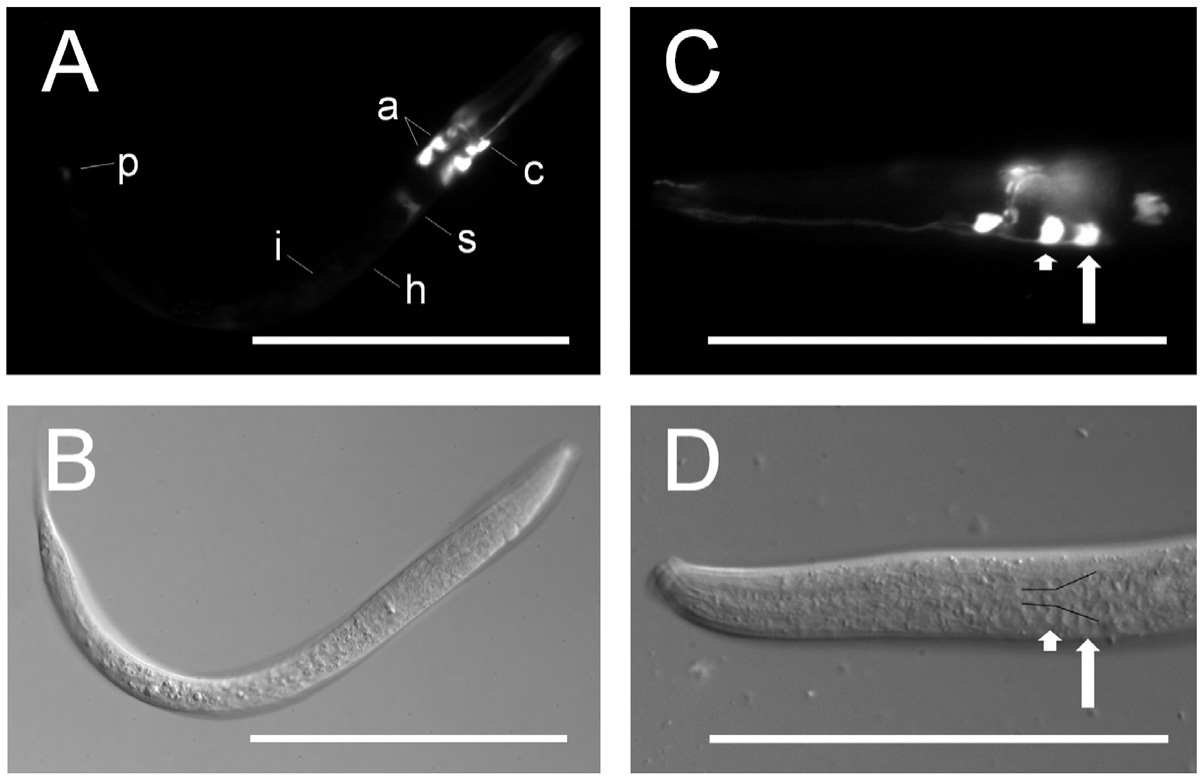
*Ce-age-1* is expressed in amphidial neurons and other tissues. Fluorescence (A,C) and DIC (B,D) images of transgenic *C. elegans* first-stage larvae expressing *Ce-age-1p::Ce-age-1(102bp) ::egfp::Ce-age-1t* from an extra-chromosomal array. (A,B) Strong expression of the EGFP reporter was present in amphidial neurons (a), a neuron or support cell anterior to the nerve ring (c), and the sphincter connecting the pharynx to the intestine (s). Weak expression was present in the intestine (i), hypodermis (h), and a phasmidial neuron (p). (C,D) EGFP reporter expression was present in the amphidial neurons AWC (short arrow) and ASJ (long arrow). Cell bodies of the amphidial neurons align just lateral to the black lines in panel D [74]. Scale bars = 100 µm.

In all six lines, we observed strong EGFP fluorescence in two pairs of amphidial neurons and their dendritic processes, a pair of inter-neurons or support cells anterior to the nerve ring, and the sphincter connecting the pharynx to the intestine (Figure 3A–D). We also noted variable expression in the hypodermis and the intestine between lines, with some lines having moderate expression in these tissues and others having little or no expression. Weak expression in a phasmidial neuron was observed in a minority of worms in each line. This led us to conclude that the main regulatory elements controlling anatomical expression of *Ce-age-1* are located in the putative promoter region and not in the 5′ coding sequence or 3′ region.

Intestinal fluorescence was often weak and punctate in all transgenic lines except BC10837, an observation consistent with auto-florescence or limited expression. Since intestinal expression in *C. elegans* is controlled by the ELT-2 transcription factor [65], [66], we searched for extended TGATAA motifs in the 5′ region of *Ce-age-1*. A single non-consensus motif at −289 bp was identified within 2 kb upstream of the *Ce-age-1* translation start site, potentially supporting our observation of weak intestinal expression. However, this observation does not preclude the possibility of distant enhancer regions that result in strong intestinal expression of *Ce-age-1* from its native locus *in vivo*.

To test the functionality of the *Ce-age-1* promoter region, we made a construct fusing the *Ce-age-1* promoter to a full-length *Ce-age-1* cDNA. We found that this construct was sufficient to fully rescue constitutive dauer arrest in two lines derived from the CY246 strain that carries the *Ce-age-1(mg44)* putative null allele. We were able to maintain these transgenic lines for more than 10 generations, indicating complete complementation. Thus, we concluded that we had identified the major tissues in which *Ce-age-1* is expressed, providing a consistent basis for comparison with *Ss-age-1*.

**Figure 4:**
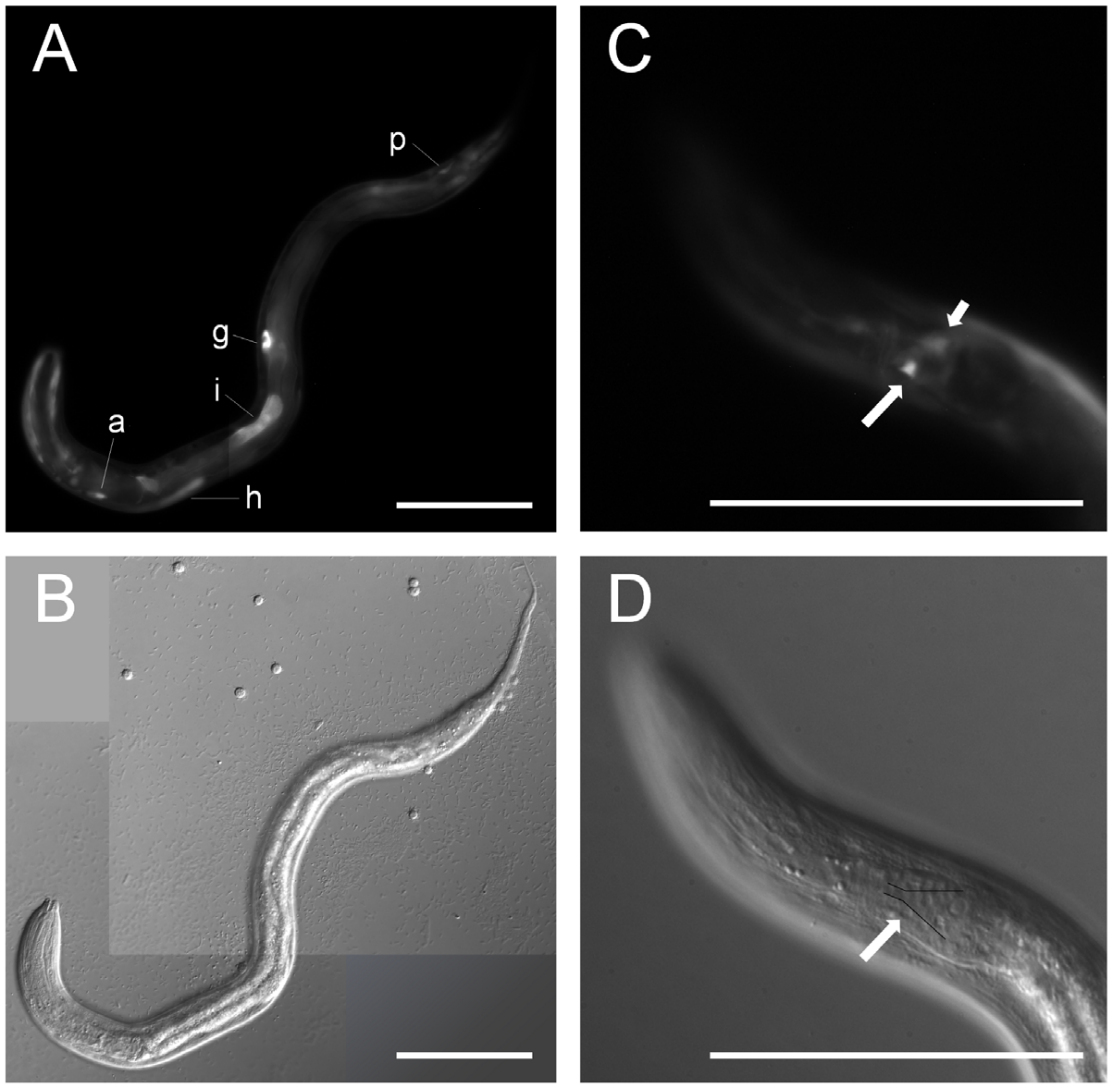
*Ss-age-1* is expressed in amphidial neurons, the intestine, and other tissues. Fluorescence (A,C) and DIC (B,D) images of transgenic *S. stercoralis* post-free-living first-stage larvae expressing *Ss-age-1p::egfp::Ss-era-1t* from an extra-chromosomal array. (A,B) Expression of the EGFP reporter was present in the intestine (i), gonadal primordium (g), amphidial/head neuron (a), hypodermis (h), and phasmidial/tail neuron (p). (C,D) Expression of the EGFP reporter was present in an amphidial neuron (long arrow), with positional homology to AWC in *C. elegans*. The other cell body of the amphidial neuron pair is out of the plane of focus (short arrow). Cell bodies of the amphidial neurons align just lateral to the black lines in panel D [74]. Scale bars = 100 µm.

To determine the anatomical expression pattern of *Ss-age-1*, we transformed parental free-living *S. stercoralis* females with a construct fusing 1.3 kb of *Ss-age-1* 5′ region to *egfp*, along with an *Ss-act-2p*::*mRFPmars* co-injection marker, as previously described [7]. Injected females were paired with *S. stercoralis* males, and their post-free-living L1 progeny were screened for fluorescence. Of the more than 1,000 F1 larvae screened, 55 transgenic larvae expressing EGFP were observed. Each transgenic L1 was imaged at 400x magnification and scored for fluorescence in several tissues (Table 1). Strong EGFP expression was most frequently observed in the anterior intestine, gonadal primordium, amphidial/head neurons, and phasmidial/tail neurons (Figure 4A–D). Consistent with the observed strong intestinal expression, a search of the 1.3 kb region upstream of the *Ss-age-1* translational start site for ELT-2 recognition motifs [66] revealed two consensus TGATAA motifs 136 bp and 630 bp upstream of the start site.

**Table 1:** Sites of *Ss-age-1* expression in transgenic *S. stercoralis* post-free-living first-stage larvae.

	Intestine	Gonadal Primorium	Head Neuron(s)	Tail Neuron(s)	Sheath/Socket Cell(s)	Hypodermis	Pharynx	Other Cell Body	Total
Expt 1 n, (%)	11 (73%)	11 (73%)	8 (53%)	4 (27%)	5 (33%)	6 (40%)	6 (40%)	3 (20%)	15
Expt 2 n, (%)	15 (79%)	7 (37%)	7 (37%)	4 (21%)	8 (42%)	7 (37%)	4 (21%)	2 (11%)	19
Expt 3 n, (%)	15 (71%)	12 (57%)	5 (24%)	4 (19%)	5 (24%)	6 (29%)	6 (29%)	2 (10%)	21
Total n, (%)	41 (75%)	30 (55%)	20 (36%)	12 (22%)	18 (33%)	19 (35%)	16 (29%)	7 (13%)	55

Since specific amphidial neurons are important in the arrest and recovery of both *C. elegans* dauer larvae [67], [68] and *S. stercoralis* L3i [55], [69], [70], we sought to identify the amphidial neurons in which *Ce-age-1* and *Ss-age-1* are expressed. Examination of transgenic *C. elegans* L1 and adult hermaphrodites revealed expression of *Ce-age-1* in the AWC and ASJ amphidial neuron pairs (Figure 3C–D). AWC neurons are important in thermotaxis [71] as well as chemotaxis to volatile odorants [72], [73]. ASJ neurons play an important role in regulating dauer formation [68] as well as dauer recovery [25], [67]. Additionally, ASJ neurons are sites of expression of *daf-28* [23] and *ins-6* [25], both encoding agonistic insulin-like peptides.

A similar examination of transgenic *S. stercoralis* post-free-living L1 was limited by the number of worms expressing the construct in amphidial neurons. However, using neuronal maps for both *Caenorhabditis spp.* [74] and *P. trichosuri* [75], we were able to locate expression in an amphidial neuron pair that we regard as the positional homolog of the AWC amphidial neuron pair in *C. elegans* (Figure 4C–D). This does not preclude the possibility of *Ss-age-1* expression in other amphidial neurons, including ASJ; however, the current limitations of transgenesis in *S. stercoralis* prevented us from pursuing this in the present study.

We conclude that although the anatomical expression patterns of *Ce-age-1* and *Ss-age-1* are not completely consistent, expression in important sites of IIS regulation, including amphidial neurons, are conserved between the two species (Figure 3C and Figure 4C). Conservation of expression in these tissues, which sense and direct responses to environmental cues in both species, is consistent with the hypothesis that the function of AGE-1 in directing dauer and L3i development is conserved between *C. elegans* and *S. stercoralis*. Furthermore, the *C. elegans* intestine is an important endocrine tissue [76] and expresses antagonistic insulin-like ligands [22]. Strong expression of *Ss-age-1* in the intestine (Figure 4A and Table 1) suggests that the intestine may also be a site of IIS regulation during L3i development in *S. stercoralis*.

### PI3 Kinase Inhibition Blocks *S. stercoralis* L3i Activation

Previous work in *C. elegans* has demonstrated that pharmacological inhibition of IIS, using the PI3 kinase inhibitor LY294002, leads to dauer arrest under well-fed conditions [77]. This work was extended to clade V parasitic nematodes, the same clade as *C. elegans*, by Brand and Hawdon, who demonstrated that LY294002 could inhibit feeding of *Ancylostoma caninum* and *A. ceylanicum* L3i [78]. LY294002 has a similar effect on larval development in another clade V nematode, *Nippostrongylus brasiliensis* [79]. However, it has never been demonstrated that inhibition of IIS signaling prevents resumption of feeding by infective larvae in any other clade of parasitic nematode in phylum Nematoda, in which animal parasitism is thought to have evolved independently at least four times [58].

To determine whether IIS regulates activation of clade IV *S. stercoralis* L3i under host-like conditions, we performed an *in vitro* L3i activation assay [55], [56]. Host-like conditions included incubation in DMEM, supplemented with 10% canine serum and 12.5 mM reduced glutathione, for 24 hours at 37°C and 5% CO_2_. Resumption of feeding, a hallmark of activation, was assessed by the ingestion of FITC dye into the pharynx.

**Figure 5:**
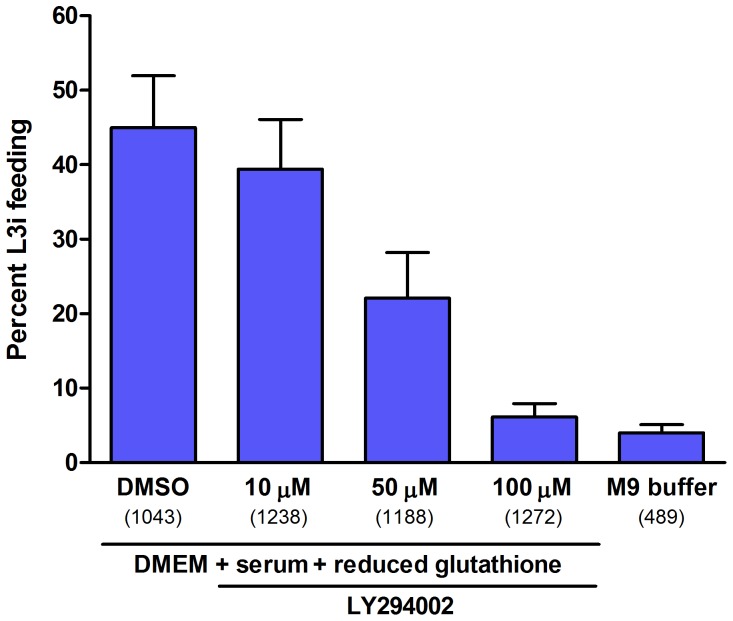
*S. stercoralis* L3i activation is attenuated by the PI3 kinase inhibitor LY294002. *In vitro* activation of *S. stercoralis* L3i under host-like culture conditions by incubation in DMEM, 10% canine serum, and 12.5 mM reduced glutathione for 24 hours at 37°C and 5% CO_2_. The percentage of L3i that resumed feeding, a hallmark of activation, was scored by ingestion of FITC into the pharynx. Conditions included DMSO (carrier) positive control and M9 buffer negative control. The PI3 kinase inhibitor LY294002 was evaluated at 100 µM, 50 µM, and 10 µM, with each condition compared to the DMSO control using a logistic regression analysis. Error bars represent +1 SEM and parenthetical integers show the total number of L3i evaluated for each condition.

We assessed the percentage of L3i feeding in three concentrations of LY294002, 10–100 µM, and compared this to a DMSO (carrier) positive control. We observed a significant, dose-dependent reduction in L3i activation in response to LY294002 (Figure 5). Logistic regression analysis revealed a 90% decrease (odds ratio: 0.10, 95% confidence interval: 0.08–0.13) in the odds of resumption of feeding for L3i at 100 µM, a 67% decrease (odds ratio: 0.33, 95% confidence interval: 0.28–0.40) in the odds of resumption of feeding for L3i at 50 µM, and a 25% decrease (odds ratio: 0.75, 95% confidence interval: 0.63–0.89) in the odds of resumption of feeding for L3i at 10 µM, all in comparison to the DMSO positive control. All odds ratios were significant at conventional statistical probabilities (p<0.001). At 100 µM of the PI3 kinase inhibitor, resumption of feeding by L3i was nearly inhibited to the level observed in the M9 buffer negative control. These data support the hypothesis that IIS signaling through the PI3 kinase, composed of *Ss-age-1* and *Ss-aap-1*, is necessary for resumption of L3i development in *S. stercoralis* upon encountering a host.

To our knowledge, these data demonstrate, for the first time, that PI3 kinase function and IIS is important in L3i activation in nematodes outside of clade V. This suggests that IIS may regulate activation of L3i in other parasitic nematode species upon encountering their respective hosts. Future studies may provide additional insight into the role of IIS in modulating L3i activation.

### Implications for the ‘Dauer Hypothesis’ and Future Directions

We have previously demonstrated that the *S. stercoralis* ortholog of the *Ce*-DAF-16 forkhead transcription factor is required for L3i arrest [6]. This is consistent with the function of *Ce*-DAF-16, which translocates to the nucleus when IIS is diminished under dauer-promoting conditions [30]. In this study, we have presented evidence that the *S. stercoralis* homolog of *Ce*-AGE-1 regulates L3i activation. This is also consistent with the function of *Ce*-AGE-1, which mediates an increase in IIS when dauer larvae encounter favorable conditions and resume development [25], [80]. Together, these data suggest that an IIS pathway, similar to that in *C. elegans*, mediates both developmental arrest of *S. stercoralis* L3i as well as activation of these infective larvae when a suitable host is encountered. Thus, we find the “dauer hypothesis" to be a relevant and informative framework for investigating the mechanisms governing L3i development in the parasitic nematode *S. stercoralis*.

Future progress towards defining the role of *Ss-age-1* in the development of *S. stercoralis* L3i will require new or enhanced functional genomic tools. Knock-down of the *Ss-age-1* message using RNA interference, which has previously been of limited utility in many parasitic nematodes including *S. stercoralis* [81]–[83], could provide an excellent means of assessing the role of *Ss-age-1* throughout the life cycle. Previously, gene function in parasitic nematodes has also been inferred from heterologous complementation of mutations in *C. elegans,* an approach we have taken to assess the function of *Ss-daf-16* [84]. However, using established methods [34], [84], we have been unable to express *Ss-age-1* sequences in the *C. elegans* CY246 strain to ascertain heterologous complementation. Future studies may reveal the mechanism that accounts for this lack of expression and allow us to pursue this line of experimentation.

Improvements in *S. stercoralis* transgenesis would also provide several more definitive means of assessing the function of *Ss-age-1*. Currently, we are able to observe effects of transgene expression in only small numbers of transiently transformed *S. stercoralis* in the post free-living generation [6]. Inducible expression of dominant loss- or gain-of-function constructs targeting *Ss-age-1* or other IIS components, preferably in stable transgenic lines, would allow us to interrogate functions of specific genes and to assess the epistatic relationships between components of the IIS pathway in regulating development of *S. stercoralis* L3i. Similar constructs could also be used to determine the role of *Ss-age-1* in homogonic versus heterogonic development of *S. stercoralis* post-parasitic L1. Our studies to elucidate the role of *Ss-age-1* in post-parasitic development using the PI3 kinase inhibitor LY294002 have thus far been uninterpretable due, we believe, to our inability to procure developmentally uncommitted L1 [70]. As we establish more robust methods, including the ability to generate stable transgenic lines and undertake conditional transgene expression, we will address additional questions regarding the role of *Ss-age-1* and of IIS generally in *S. stercoralis* development. A greater understanding of the role of IIS in L3i development may lead to novel control strategies as well as new treatments for strongyloidiasis and other diseases caused by parasitic nematodes.

## Supporting Information

Figure S1 **Ss-AAP-1 is a PI3 kinase accessory/regulatory subunit.** Protein alignment of PI3 kinase accessory/regulatory subunits. The two Scr homology 2 (SH2) domains (grey bars) have the highest conservation of residues (black highlights) between all sequences. Abbreviations: *Strongyloides stercoralis* (Ss), *Strongyloides ratti* (Sr), *Brugia malayi* (Bm), *Ascaris suum* (As), *Trichinella spiralis* (Ts), *Caenorhabditis elegans* (Ce), *Homo sapiens* (Hs), and *Drosophila melanogaster* (Dm). Accession numbers listed in Methods.
(TIF)

Table S1 **Primer names and sequences.**
(DOC)

Dataset S1 **Predicted protein sequences for** ***Strongyloides ratti age-1*** **and** ***aap-1*** **homologs.**
(DOC)

Text S1 **Supplemental Methods - isolation of** ***S. stercoralis*** **developmental stages.**
(DOC)
