# Supplementary figures and images for: Strongyloides stercoralis age-1: A Potential Regulator of Infective Larval Development in a Parasitic Nematode
Source: PLoS One. 2012 Jun 6;7(6):e38587. doi: 10.1371/journal.pone.0038587 (PMC3368883; doi:10.1371/journal.pone.0038587)

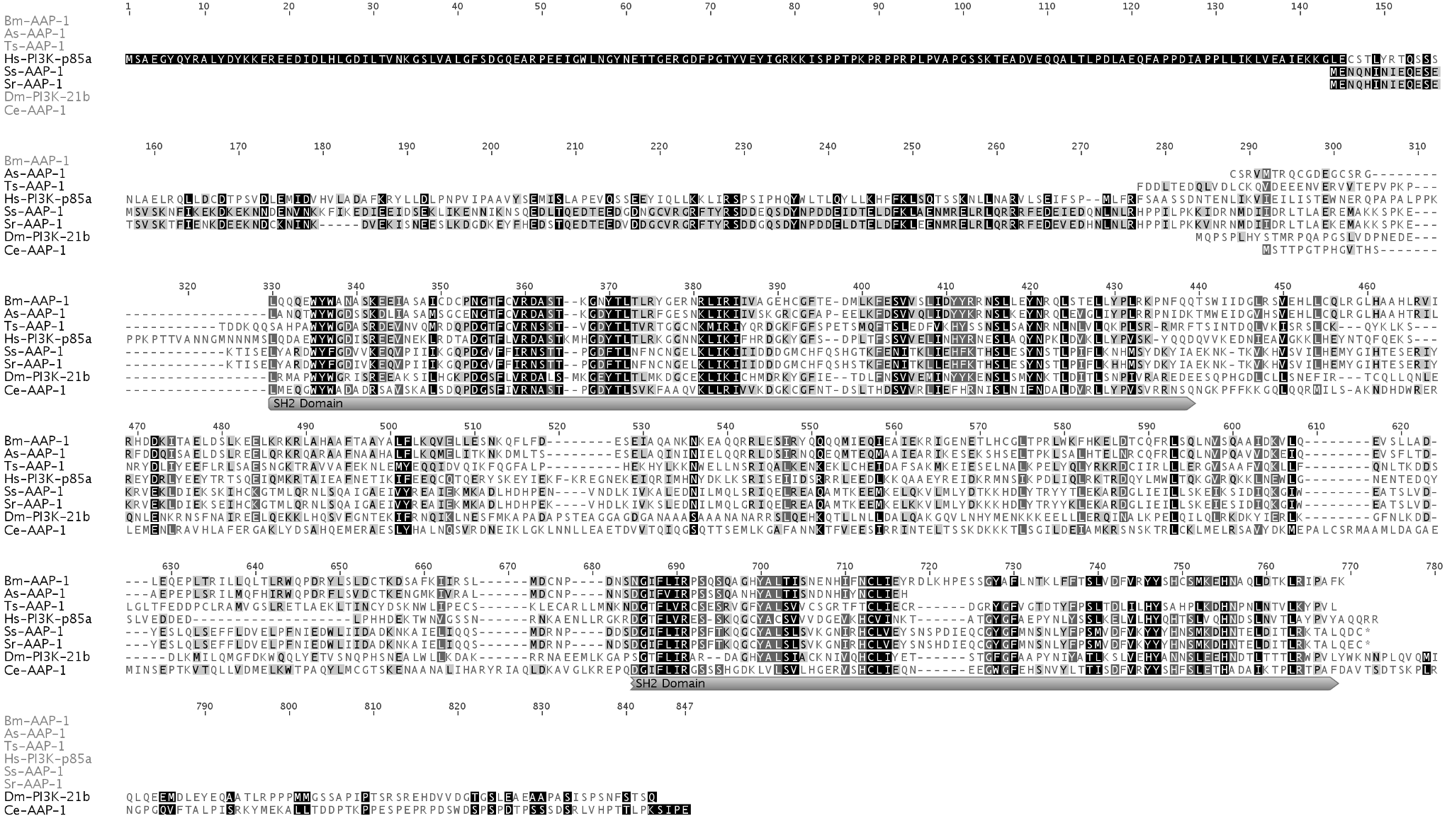

Supplement: Figure S1 — Ss-AAP-1 is a PI3 kinase accessory/regulatory subunit. Protein alignment of PI3 kinase accessory/regulatory subunits. The two Scr homology 2 (SH2) domains (grey bars) have the highest conservation of residues (black highlights) between all sequences. Abbreviations: Strongyloides stercoralis (Ss), Strongyloides ratti (Sr), Brugia malayi (Bm), Ascaris suum (As), Trichinella spiralis (Ts), Caenorhabditis elegans (Ce), Homo sapiens (Hs), and Drosophila melanogaster (Dm). Accession numbers listed in Methods. (TIF) [file pone.0038587.s001.tif]
