# Supplementary material for: Strongyloides stercoralis age-1: A Potential Regulator of Infective Larval Development in a Parasitic Nematode
Source: PLoS One. 2012 Jun 6;7(6):e38587. doi: 10.1371/journal.pone.0038587 (PMC3368883; doi:10.1371/journal.pone.0038587)
Supplement: Table S1 — Primer names and sequences. (DOC) [file pone.0038587.s002.doc]

**Table S1: Primer names and sequences.**

| **Primer Name** | **Primer Sequence (5’ to 3’)** |
| --- | --- |
| **Ss-age-1-11F** | CTTCTTTAACAGGTTATTGCGTTGCAACG |
| **Ss-age-1-17R** | ATCCAATTGCTACTCCAATATCTTGTTC |
| **Ss-age-1-20F** | GGATGCTTTTTGGAAATCAATGGGTTATG |
| **Ss-age-1-20R** | GCAATCATTGCAAAAAGGTAATTTTGTATTGG |
| **Ss-age-1-23FattB4** | ggggacaactttgtatagaaaagttgggGCTAATCGTACAAAATTACTTTCAGGTG |
| **Ss-age-1-23RattB1r** | ggggactgcttttttgtacaaacttggATCCTTTTAATGAAAAGGAAGTTATAAATTG |
| **Ss-age-1-24FattB1** | ggggacaagtttgtacaaaaaagcaggctccATGAATGTTCCAACATCATCAAATAAAC |
| **Ss-age-1-24RstopattB2** | ggggaccactttgtacaagaaagctgggtcTTACATATGTTTAACTGAATGGAAAAACC |
| **Ss-age-1-EGFP-F** | caatttataacttccttttcattaaaaggatATGAGTAAAGGAGAAGAACTTTTC |
| **Ss-age-1-EGFP-R** | GAAAAGTTCTTCTCCTTTACTCATatccttttaatgaaaaggaagttataaattg |
| **EGFP-Ss-era-1-F** | CATGGACGAACTATACAAATGAGATAActaggaatttattttgataagtctcg |
| **EGFP-Ss-era-1-R** | cgagacttatcaaaataaattcctagTTATCTCATTTGTATAGTTCGTCCATG |
| **Ssaap1-2R** | CCATACATTTCATGAAGAATGACTGAGACATG |
| **Ssaap1-3R** | CCATGAGATTGGAAGTGACACATTCCATC |
| **Ssage1RT-3F** | TGAATGTTCCAACATCATCAAA |
| **Ssage1RT-3R** | TTGATTTAAATGGATCAGGAGGT |
| **Ssact2RT-2F** | AGAAGCTATGTTCCAACCATCA |
| **Ssact2RT-2R** | ATTGTGGTACCTCCGGAAAG |
| **SsgapdhRT-2F** | CATGGAAGATTCAAAGGATCTG |
| **SsgapdhRT-2R** | TCAGCTGGGTCTCTACAGTTG |
| **Ce-age-1-EGFP-F** | caagctttcattttaagattttaagATGAGTAAAGGAGAAGAACTTTTC |
| **Ce-age-1-EGFP-R** | GAAAAGTTCTTCTCCTTTACTCATcttaaaatcttaaaatgaaagcttg |
| **Ex10837-EGFP-F** | acggaactcccgacgtatcATGAGTAAAGGAGAAGAACTTTTC |
| **Ex10837-EGFP-R** | GAAAAGTTCTTCTCCTTTACTCATgatacgtcgggagttccgt |
| **EGFP-Ceage1t-F** | GCATGGACGAACTATACAAATGAaacctctgttatctaataatataacacattc |
| **EGFP-Ceage1t-R** | gaatgtgttatattattagataacagaggttTCATTTGTATAGTTCGTCCATGC |
| **Ceage1p-cDNA-F** | caagctttcattttaagattttaagATGTCTATGGGACGAAGCCCCTC |
| **Ceage1p-cDNA-R** | GAGGGGCTTCGTCCCATAGACATcttaaaatcttaaaatgaaagcttg |
| **Ceage1-Cat-F** | CCTCCGTGGAAATGAAGAGCACatcaagatcatcacccgacaag |
| **Ceage1-Cat-R** | cttgtcgggtgatgatcttgatGTGCTCTTCATTTCCACGGAGG |
| **Ceage1-unc54-F** | CCACGCAGTCAAACACTACTGAgataagagctccgcatcggccg |
| **Ceage1-unc54-R** | cggccgatgcggagctcttatcTCAGTAGTGTTTGACTGCGTGG |
