# Supplementary material for: Strongyloides stercoralis age-1: A Potential Regulator of Infective Larval Development in a Parasitic Nematode
Source: PLoS One. 2012 Jun 6;7(6):e38587. doi: 10.1371/journal.pone.0038587 (PMC3368883; doi:10.1371/journal.pone.0038587)
Supplement: Dataset S1 — Predicted protein sequences for Strongyloides ratti age-1 and aap-1 homologs. (DOC) [file pone.0038587.s003.doc]

**Dataset S1: Predicted protein sequences for *Strongyloides ratti age-1* and *aap-1* homologs.**

Predicted protein sequences for the *Strongyloides ratti* homologs of the *C. elegans* PI3 kinase subunits AGE-1 and AAP-1 (* denotes putative stop codon).

***Sr*-AGE-1:**

MNIPTSTNKHVHIQEPPDPFKSKKRHEEHKRKANDAKYPSHKNVERNVNYYQRFNKKSETRDFYNDIKNKTLYDNVKEMLEMLNSDYKMSDYVNLIGKYALKNNVWKNFVKYSKIKEFSLTDREYVEFDILLPDGFLITINISARATLEQLKKEVFYQANKFIFNKNLLPIQNYLFAMIAANGSKENIYDESCQLFVYKLKSPLLVLHQPSENVVEKKLEQDIGVAIGFPIDELDQKISYEAKLFRVSLFEFCVQTISERCCSGNGHYAFFEDNILELEYKLTPKIQNKLDEKNMLTRVYYRSYEDEKNELDTKCTCIDIYNVISKDKETGVVSIMTIQNLIEYSLNQLKNMGINIKEEEKDFVLQIVGKKIYLTKENIPLTSFEYIRSSFDNDNIPLLIMCRKNLIYNNLPPYVNMHVPYYVRGNRKQKEITHLHLHEDHGDIQYLWEFEDDFKFELDTAGNVSVYDSEQKIFVRVALTVGRHILAQKDSTHKSINDPRWRGYKMNLGYYMKDIPPAAQLSFALVSTVKKKNGKSENEVLGWCNLRLFDYKHRLIQGRKTLYLHNPGPQVEDNYINPSGPESMNIQKGSHSRIVVYFKDYSVKNRKIHIKYPEMSKIKQYASIIKNNPRISDEDKAIDPNFVKSEDVKKLKKYLKYLDGTQLSEDDQYYLWKHREYICTHLPNLLVVISDCKRIWKSREHVAQFYELLTRWGNISVEAAIELLDNRQRDCEVRKFAVDILDKWLDDERFKLFMMHLIQGIKYEPYYDNPLAVMLIRRALLNYQIAHHLFWLLRGELEQEIGSSESVNGKECRLTGDNNIEGKLTLPVVRCTIMLECLLRANVRHIGPVIKQVRMVNELFKISNEIKNNTSKENNTKLLQRKLKKVIHEMEYVESPLNPIILLGELCVEECRVLSSAKQPLKLVWSNCEPLARLSKKTHQIIFKNGDDLRQDMLTLQVMKIMDAFWKSMGYDFCMSIYEVLPMGYNIGLINVVQKCVTLFEIQTNEKKRSMPLSMETACINKWLRKYNDETKLYLEAVDKFTASLTGYCVATYILGIKDRHQDNIMVRKDGRMFHIDFGHILGHTKTKLGINRDRTPFILTNHFLFVITKGRCQFKNDHDIIKFRENCRKAFLILHDHSRLFISLFRMMLSMGLPECSTQEDLNYLKLSLMAGFDKDIAAIQFDNIFDEVIKSDLSTKTNWFFHSVKHM*

***Sr*-AAP-1:**

MENQHINIEQESETSVSKTFIENKDEEKNDCKNINKDVEKISNEESLKDGDKEYFHEDSTQEDTEEDVDDGCVRGRFTYRSDDGQSDYNPDDELDTELDFKLEENMRELRLQRRRFEDEVEDHNLNLRHPPILPKKVNRNMDIIDRLTLAEKEMAKKSPKEKTISELYARDWYFGDIVKEQVPIIIKGQPDGVFFIRNSTTPGDFTLNFNCNGELKLIKIIIDDDGMCHFQSHSTKFENITKLLEHFKTHSLESYNSTLPIFLKHHMSYDKYIAEKNKTKVKHVSVILHEMYGIHTESERIYKRVEKLDIEKSEIHCKGTMLQRNLSQAIGAEIVYREAIEKMKADLHDHPEKVHDLKIVKSLEDNILMQLGRIQELREAQAMTKEEMKELKKVLMLYDKKKHDLYTRYYKLEKARDGLIEILLSKEIESIDIQKGIWEATSLVDYESLQLSEFFLDVELPFNIEDWLIIDADKNKAIELIQQSMDRNPNDSDGIFLIRPSFTKQGCYALSLSVKGNIRHCLVEYSNSHDIEQCGYGFMNSNLYFPSMVDFVKYYYHNSMKDHNTELDITLRKTALQEC*
