# Supplementary material for: Strongyloides stercoralis age-1: A Potential Regulator of Infective Larval Development in a Parasitic Nematode
Source: PLoS One. 2012 Jun 6;7(6):e38587. doi: 10.1371/journal.pone.0038587 (PMC3368883; doi:10.1371/journal.pone.0038587)
Supplement: Text S1 — Supplemental Methods - isolation of S. stercoralis developmental stages. (DOC) [file pone.0038587.s004.doc]

**Text S1: Supplemental Methods - Isolation of *S. stercoralis* Developmental Stages**

**Post-parasitic first-stage larvae (PP L1)**

Post-parasitic L1 from the *S. stercoralis* PV001 line, which has >95% heterogonic development under standard culture conditions, were isolated. Larvae were migrated from fresh feces using the Baermann technique, with a 28-29°C water temperature for two hours. Four 50 ml aliquots were drained, centrifuged at room temperature for 10 minutes, and the supernatants removed. Pelleted debris were combined and mixed with an equal volume of liquid, 1% low melting temperature agarose (Lonza, Basel, Switzerland) at ~30°C. The suspension was transferred to a petri dish and allowed to solidify, first on 4°C glass and then at room temperature. Subsequently, 14 ml of BU buffer was added, and the dish was placed at 25-29°C for two hours, allowing larvae to migrate into the liquid. Any precociously developing L3i were removed from the liquid, which was then transferred to a 15 ml tube, centrifuged at room temperature for 10 minutes, and the supernatant was removed. The larval pellet was transferred to a 1.5 ml tube, centrifuged again, and the supernatant was removed. Larvae were mixed with 200 μl of TRIzol reagent and snap frozen in liquid nitrogen.

**Post-parasitic third-stage larvae (PP L3) heterogonically developing to free-living adults**

*S. stercoralis* PV001 line larvae were migrated from charcoal coprocultures, incubated for 24 hours at 21°C, using the Baermann technique, with a 28-29°C water temperature for one hour. One 40 ml aliquot was removed, and worms were allowed to settle at 1 *g* for 10 minutes. Larvae were mixed with an equal volume of liquid, 1% low melting temperature agarose at ~30°C. The suspension was transferred to a petri dish and allowed to solidify, first on 4°C glass and then at room temperature. Subsequently, 14 ml of BU buffer was added, and the dish was placed at 25-29°C for one hour, allowing larvae to migrate into the liquid. Approximately third-stage larvae, identified by overall length and an elongated gonadal primordium (late L2 or L3) but without development of gonadal arms (L4), were removed to a 1.5 ml tube with a pipette. Larvae with a visible spicule, developing to adult males, were not selected, thus enriching the population for larvae developing to free-living females. Larvae were allowed to settle at 1 *g* for 10 minutes, and the supernatant was removed. Larvae were mixed with 200 μl of TRIzol reagent and snap frozen in liquid nitrogen.

**Free-living females (FL Females)**

*S. stercoralis* PV001 line adult free-living worms were migrated from charcoal coprocultures, incubated for 48 hours at 21°C, using the Baermann technique, with a 28-29°C water temperature for one hour. One 30 ml aliquot was removed, and worms were allowed to settle at 1 *g* for 10 minutes. Worms were mixed with an equal volume of liquid, 1% low melting temperature agarose at ~30°C. The suspension was transferred to a petri dish and allowed to solidify, first on 4°C glass and then at room temperature. Subsequently, 14 ml of BU buffer was added, and the dish was placed at 28°C for 30 minutes, allowing adult worms to migrate into the liquid. Free-living females with 2-10 eggs per gonadal arm were removed to a 1.5 ml tube with a pipette, allowed to settle at 1 *g* for 10 minutes, and the supernatant was removed. Females were then mixed with 200 μl of TRIzol reagent and snap frozen in liquid nitrogen.

**Post-free-living first-stage larvae (PFL L1)**

*S. stercoralis* PV001 line larvae were migrated from charcoal coprocultures, incubated for three days at 21°C, using the Baermann technique, with a 29°C water temperature for two hours. Four 50 ml aliquots were drained and allowed to settle at 1 *g* for 6-8 minutes. Free-living adults were removed from the bottom of each tube. The remaining suspension of larvae was centrifuged at room temperature for 10 minutes, and the supernatants were removed. Pelleted larvae were combined and mixed with an equal volume of liquid, 1% low melting temperature agarose at ~30°C. The suspension was transferred to a petri dish and allowed to solidify, first on 4°C glass and then at room temperature. Subsequently, 14 ml of BU buffer was added, and the dish was placed at 29°C for two hours, allowing larvae to migrate into the liquid. All remaining free-living adults were removed with a pipette, leaving only post-free-living L1. The larval suspension was transferred to a 15 ml tube, centrifuged for 10 minutes, and the supernatant was removed. The larval pellet was transferred to a 1.5 ml tube, centrifuged again, and the supernatant was removed. Larvae were mixed with 200 μl of TRIzol reagent and snap frozen in liquid nitrogen.

**Infectious third-stage larvae (L3i)**

*S. stercoralis* PV001 line larvae were migrated from charcoal coprocultures, incubated for 8-10 days at 21°C or 7 days at 25°C, using the Baermann technique, with a 27-30°C water temperature for one hour. One 40 ml aliquot was removed, centrifuged for 3 minutes, and the supernatant was removed. L3i were then washed twice in deionized water, and the supernatant was removed. L3i were mixed with an equal volume of liquid, 1% low melting temperature agarose at ~30°C. The suspension was transferred to a petri dish and allowed to solidify, first on 4°C glass and then at room temperature. Subsequently, 14 ml of BU buffer was added, and the dish was placed at 28°C for one hour, allowing larvae to migrate into the liquid. The L3i suspension was transferred to a 15 ml tube, centrifuged for 10 minutes, and the supernatant was removed. L3i in 200 μl aliquots were divided among 1.5 ml tubes, and each aliquot of L3i was mixed with 200 μl of TRIzol reagent and snap frozen in liquid nitrogen.

**Parasitic females (P Females)**

*S. stercoralis* PV001 line L3i for experimental infections were isolated from seven day-old cultures incubated at 25°C by the Baermann technique, with a 28°C water temperature for one hour. One 40 ml aliquot was removed, centrifuged for five minutes at room temperature, and the supernatant was removed. L3i were then washed once more in deionized water and the supernatant was removed. L3i were mixed with an equal volume of liquid, 1% low melting temperature agarose at ~30°C. The suspension was transferred to a petri dish and allowed to solidify, first on 4°C glass and then at room temperature. Subsequently, 14 ml of BU buffer was added, and the dish was placed at 29°C for one hour, allowing larvae to migrate into the liquid. The L3i suspension was transferred to a 15 ml tube, centrifuged for five minutes, and the supernatant was removed. L3i were then suspended in phosphate buffered saline (PBS) at a concentration of 4,000 L3i per 200 μl.

Mongolian gerbils were experimentally infected with 4,000 L3i and sacrificed three weeks after infection. The gastrointestinal tract was removed and hung separately for each gerbil in graduated cylinders with DMEM, supplemented with gentamycin, for three hours at 37°C. Parasitic females were separated from the intestinal mucosa by repeatedly pipetting worms into fresh medium and removing debris. Cleaned worms were allowed to settle at 1 *g* for 10 minutes and the supernatant was removed. Parasitic females from infections of single gerbils were then mixed with 200 μl of TRIzol reagent and snap frozen in liquid nitrogen.
